# The Marine Gastropod *Crepidula fornicata* Remains Resilient to Ocean Acidification Across Two Life History Stages

**DOI:** 10.3389/fphys.2021.702864

**Published:** 2021-08-25

**Authors:** Christopher L. Reyes-Giler, Brooke E. Benson, Morgan Levy, Xuqing Chen, Anthony Pires, Jan A. Pechenik, Sarah W. Davies

**Affiliations:** ^1^Department of Biology, Boston University, Boston, MA, United States; ^2^Department of Biology, Tufts University, Medford, MA, United States; ^3^Department of Biology, Dickinson College, Carlisle, PA, United States

**Keywords:** gastropod, *Crepidula fornicata*, plasticity, carryover effects, gene expression, life history, ocean acidification

## Abstract

Rising atmospheric CO_2_ reduces seawater pH causing ocean acidification (OA). Understanding how resilient marine organisms respond to OA may help predict how community dynamics will shift as CO_2_ continues rising. The common slipper shell snail *Crepidula fornicata* is a marine gastropod native to eastern North America that has been a successful invader along the western European coastline and elsewhere. It has also been previously shown to be resilient to global change stressors. To examine the mechanisms underlying *C. fornicata’s* resilience to OA, we conducted two controlled laboratory experiments. First, we examined several phenotypes and genome-wide gene expression of *C. fornicata* in response to pH treatments (7.5, 7.6, and 8.0) throughout the larval stage and then tested how conditions experienced as larvae influenced juvenile stages (i.e., carry-over effects). Second, we examined genome-wide gene expression patterns of *C. fornicata* larvae in response to acute (4, 10, 24, and 48 h) pH treatment (7.5 and 8.0). Both *C. fornicata* larvae and juveniles exhibited resilience to OA and their gene expression responses highlight the role of transcriptome plasticity in this resilience. Larvae did not exhibit reduced growth under OA until they were at least 8 days old. These phenotypic effects were preceded by broad transcriptomic changes, which likely served as an acclimation mechanism for combating reduced pH conditions frequently experienced in littoral zones. Larvae reared in reduced pH conditions also took longer to become competent to metamorphose. In addition, while juvenile sizes at metamorphosis reflected larval rearing pH conditions, no carry-over effects on juvenile growth rates were observed. Transcriptomic analyses suggest increased metabolism under OA, which may indicate compensation in reduced pH environments. Transcriptomic analyses through time suggest that these energetic burdens experienced under OA eventually dissipate, allowing *C. fornicata* to reduce metabolic demands and acclimate to reduced pH. Carry-over effects from larval OA conditions were observed in juveniles; however, these effects were larger for more severe OA conditions and larvae reared in those conditions also demonstrated less transcriptome elasticity. This study highlights the importance of assessing the effects of OA across life history stages and demonstrates how transcriptomic plasticity may allow highly resilient organisms, like *C. fornicata*, to acclimate to reduced pH environments.

## Introduction

Rising atmospheric carbon dioxide (CO_2_) concentrations, resulting from anthropogenic emissions, are causing substantial increases in the acidity of the world’s oceans (Bigg et al., 2003; IPCC, 2013). This process, known as ocean acidification (OA), involves carbonic acid formed by the hydrolysis of atmospheric CO_2_ in seawater dissociating into bicarbonate [HCO_3_^–^] and hydrogen ions [H^+^], lowering seawater pH (Orr et al., 2005). Average ocean pH has declined by 0.13 pH units since 1765 and is expected to decrease a further 0.3–0.4 pH units by 2100, corresponding to an atmospheric CO_2_ concentration of 800–1000 ppm (IPCC, 2001; Orr et al., 2005). The negative effects of OA on the phenology, recruitment, and community interactions of organisms across diverse geographical regions are expected to increase as climatic conditions continue to shift (Walther et al., 2002). Additionally, nearshore ecosystems (e.g., estuaries and intertidal zones), which are characterized by substantial daily fluctuations in salinity, temperature, and pH are likely to experience even greater negative impacts in the future (Diederich and Pechenik, 2013; Waldbusser and Salisbury, 2014; Baumann et al., 2015; Pacella et al., 2018).

The negative effects of OA on marine mollusk physiology have been well documented [Vargas et al., 2013; Dineshram et al., 2015; Waldbusser et al., 2016; Maboloc and Chan, 2017; reviewed in Strader et al. (2020)]. As acidity increases, excess H^+^ recombines with carbonate to form bicarbonate. This reduction in the availability of carbonate ions, which are required by mollusks and other calcifying organisms for shell formation, has consequences for growth, morphology, and ultimately survival (Orr et al., 2005; Talmage and Gobler, 2010). Additionally, OA can reduce fertilization success, compromise induced defenses, and impair immune function (Vargas et al., 2013). Although most species exhibit reduced fitness under elevated CO_2_ concentrations (Talmage and Gobler, 2010; Guo et al., 2015), sensitivity to acidification can vary between related species and even among different populations of the same species (Talmage and Gobler, 2010; Guo et al., 2015; Noisette et al., 2016; Griffiths et al., 2019). The molecular responses of mollusks to OA are also diverse and include signatures of metabolic depression [adult blue mussels (Hüning et al., 2013), adult pearl oysters (*Pinctada fucata*) (Li et al., 2016a,b), Atlantic slippers snail larvae (Kriefall et al., 2018)], differential expression of genes associated with calcification [*Crassostrea gigas* (De Wit et al., 2018), *Heliconoides inflatus* (Moya et al., 2016), adult pearl oysters (*P. fucata*) (Li et al., 2016a,b), blue mussels (Hüning et al., 2013)], and differential regulation of genes associated with the cellular stress response [Sydney rock oyster (*Saccostrea glomerata*) (Parker et al., 2011, 2012; Dove et al., 2013), *C. gigas* (Dineshram et al., 2016; De Wit et al., 2018), Antarctic pteropods (Johnson and Hofmann, 2017), Atlantic slippers snail larvae (Kriefall et al., 2018)]. While the physiological and molecular responses to OA have been widely studied (reviewed in Strader et al. (2020)], how these responses vary across life history stages remain less explored.

Native to the eastern coast of North America, the common slipper shell snail *Crepidula fornicata* has demonstrated resilience to most environmental stressors associated with climate change (Blanchard, 1997; Noisette et al., 2016; Kriefall et al., 2018). Adults of this species have been shown to tolerate elevated *p*CO_2_ (750 μatm) for >5 months and exhibit decreased calcification rates only under the extreme *p*CO_2_ of 1400 μatm (Noisette et al., 2016). However, the early life history stages may be more vulnerable to elevated CO_2_ (Talmage and Gobler, 2010; Guo et al., 2015). Noisette et al. (2014) showed that *C. fornicata* larvae reared at reduced pH exhibited reduced shell growth and reduced mineralization and also showed signs of shell abnormalities. In addition, while there was no consistent influence of pH on larval mortality within the range of pH 7.5–8.0, reduced pH negatively impacted larval and juvenile growth rates and delayed the onset of competence for metamorphosis (Kriefall et al., 2018; Bogan et al., 2019; Pechenik et al., 2019). Short- and long-term exposure of competent larvae to reduced pH (7.5 vs. 8.0), however, did not inhibit metamorphosis in response to a natural adult-derived cue (Pechenik et al., 2019). The physiological mechanisms underlying such resilience are not well understood.

Although these studies have shed light on how this resilient mollusk copes with OA, few studies have investigated how the impacts of OA may interact across life stages in marine mollusks, despite the complexities associated with life stage transitions (Talmage and Gobler, 2011; Ross et al., 2016; Bogan et al., 2019). Here, we investigate how exposure to OA in the larval stage may influence later life stages (i.e., latent effects or carry-over effects) by assessing the physiological and transcriptomic responses of *C. fornicata* larvae reared at three pH levels (pH 7.5, 7.6, and 8.0). After metamorphosis, juveniles were raised under control conditions (pH 8.0), and the influence of pH conditions experienced as larvae (i.e., carryover effects) on juvenile growth, patterns of gene expression, and transcriptome elasticity (i.e., return to control conditions) were examined. In addition, we determined how quickly these patterns of gene expression were affected by acute pH exposure, by comparing transcriptomic responses of larvae reared at pH 8.0 (control) and pH 7.5 after 4, 10, 24, and 48 h. We demonstrate gene expression plasticity across life stages in response to OA, potentially highlighting the mechanism underlying *C. fornicata*’s resilience and capacity for acclimation to drastically altered environmental conditions.

## Materials and Methods

### Adult and Larval Collection

Brooding adults of *C. fornicata* were collected during their reproductive season in June and July 2017 from the intertidal zone in Totten Inlet, Thurston County, WA, United States and transported to the University of Washington’s Friday Harbor Laboratories (FHL) in Friday Harbor, WA. Stacks of approximately four to six adults were housed in separate, aerated 3-L glass jars containing 2 L of room temperature (∼ 23°C) unfiltered seawater, which was changed daily. Larvae hatched naturally within several days after adults were collected. Veligers were concentrated by gently siphoning the culture through a 150 μm sieve shortly after their release by the mothers. Veligers from each jar were released by a different female, and thus were considered to be separate broods. Each of the two experiments described in detail below was conducted independently on a single brood to test different questions and to control for maternal effects across broods.

### Seawater pH Manipulation and Carbonate Chemistry

Larvae were cultured in the Ocean Acidification Environmental Laboratory at FHL. Incoming seawater was filtered to 1 μm and equilibrated overnight at 20°C by bubbling with mixtures of CO_2_ and CO_2_-free air delivered by Aalborg GFC17 mass-flow controllers to achieve pH levels of 7.5, 7.6, and 8.0 (Kriefall et al., 2018). Seawater pH was measured immediately before loading into culture jars and immediately before regular seawater changes with a Honeywell Durafet pH electrode calibrated to the total scale by the cresol purple spectrophotometric method described by Dickson et al. (2007). Headspaces of culture jars were continuously ventilated with the same gas mixtures used to condition the seawater pH treatments. Temperature and salinity were measured with a YSI Pro Series 1030 m. Seawater samples were fixed with mercuric chloride and titrated to determine total alkalinity (TA) using a Mettler DL15 automated titrator calibrated to certified reference materials (Dickson laboratory, Scripps Institution of Oceanography). *p*CO_2_ and aragonite saturation state (Ω_A__r_) were estimated based on empirical measurements of pH and TA using CO_2_Sys 2.1 (Pierrot et al., 2006). Chemical and physical properties of larval culture seawater are given in Supplementary Tables 1, 2.

### Experiment I: Larval Culturing and Growth Measurements

**FIGURE 1:**
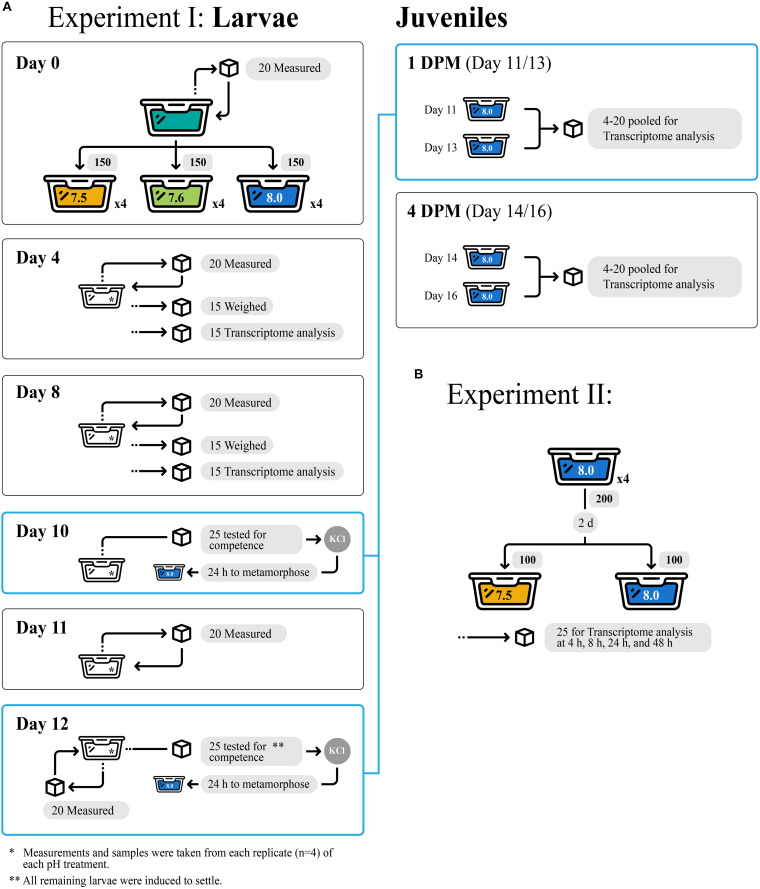
Experimental design for Experiment I **(A)** and Experiment II **(B)**, showcasing tank replicates within treatments and number of larvae/juvenile *Crepidula fornicata* measured for each trait at each timepoint (indicated with cubes).

All experimental design details of Experiment I including sample sizes are depicted in Figure 1A. One hundred and fifty larvae from a single brood were randomly assigned to each of four replicate 800-mL jars per pH treatment (7.5, 7.6, or 8.0) and reared under treatment pH conditions for 12 days (d). Every 2 d, larvae were isolated by sieving and then transferred back into jars with freshly conditioned seawater. Larvae were fed 10^4^ cells mL^–1^ of *Isochrysis galbana* (clone T-ISO) when cultures were started and at each water change, to promote maximal growth rates (Pechenik and Tyrell, 2015). Subsamples of approximately 15 larvae were collected from each replicate jar on days 4 and 8 and stored in RNA later (Thermo Fisher Scientific, Waltham, MA, United States) for gene expression profiling. It is also important to note that no larvae died in any of the three experimental treatments.

Veliger larval growth rates were quantified according to three metrics: change in shell length (μm), proportion tissue weight relative to total weight, and change in total weight (ng). Random subsamples of 20 larvae from each experimental replicate (*n* = 80 larvae/treatment) were collected soon after hatching (day 0) and 4, 8, 11, and 12 days later to document shell length growth rates (μm/day). Larvae were non-destructively imaged using a Motic camera fitted to a Leica Wild M3C dissecting microscope and then returned to their culture container. Shell lengths were measured in ImageJ (Schneider et al., 2012). Growth rates were recorded cumulatively relative to initial measurements from day of hatching (day 0). At 4- and 8-d, 15 additional larvae per replicate were fixed in 20% buffered formalin before being transferred into pre-weighed aluminum cups to assess changes in shell and tissue weight. Samples were oven dried at 60°C for at least 6 h, weighed, and then placed into a muffler furnace at approximately 500°C for 6 h to oxidize all organic material, allowing for determinations of larval tissue weights and inorganic shell weights.

### Induction and Measurement of Competence for Metamorphosis

Larval competence for metamorphosis was assayed on days 10 and 12 to estimate the impact of reduced pH on time to competence. In each experimental assay, 25 larvae per replicate (*n* = 100 larvae/treatment) were transferred to 8 mL of seawater that matched larval pH rearing conditions and were then exposed to 20 mM elevated KCl in seawater to induce metamorphosis (Pechenik and Gee, 1993). Metamorphosis was defined by the loss of the ciliated velum, and the proportion of metamorphosed larvae was assessed 6 h after induction. On day 12, all remaining larvae across all cultures were induced to metamorphose. No larvae died during metamorphosis.

### Juvenile Culturing and Growth Measurements

Metamorphosed larvae (hereafter, “juveniles”) were maintained for an additional 4 d at ambient conditions (pH 7.9–8.0, salinity 29–30 ppt, temperature 22–23°C) on a diet of T-ISO at 18 × 10^4^ cells/ml. Groups of 4–20 juveniles were subsampled for gene expression immediately following metamorphosis on day 11 or 13, approximately 24 h (1 day) after their respective induction trials, as well as 4-day post-metamorphosis (DPM), at 14- or 16-day. Collections were pooled across metamorphosis days (day 11, 13) such that all individuals at 1 DPM or 4 DPM were analyzed together regardless of actual age, in order to standardize time spent in the juvenile stage at the time of sampling. All samples were preserved in RNAlater (Thermo Fisher Scientific, Waltham, MA, United States). Mean juvenile shell length (μm) was quantified upon metamorphosis using Image J as described for larvae, and at 4 DPM using an ocular micrometer at 25 × magnification. These data were then used to calculate juvenile shell length growth rates (%/day) between 1 DPM and 4 DPM. No juvenile *C. fornicata* mortality was observed for juveniles that were reared in any of the experimental treatments as larvae.

### Experiment II: Larval Culturing

All experimental design details of Experiment II including sample sizes are depicted in Figure 1B. Here, we conducted a second study to determine how quickly larvae respond to reduced pH at the molecular level. Culturing methods for this short-term (48-h) experiment were similar to those for larvae in Experiment I described above. Briefly, larvae were sourced from a separate brood from a single female and cultured for 2 day from hatching at pH 8.0 as described above (4 replicate cultures, 200 larvae/replicate). After 2 day, larvae from each culture were equally divided into 2 new culture jars containing freshly conditioned seawater at pH 7.5 or 8.0. Larvae were fed as described above, subsampled (approximately 25 larvae from each of the 4 replicates of each pH treatment, *n* = approximately 100 larvae/treatment) after 4, 10, 24, and 48 h, and stored in RNAlater (Thermo Fisher Scientific) for gene expression profiling. No phenotypic measurements were taken for this secondary acute OA Experiment II, however, no mortality was observed.

### Statistical Analyses of Phenotypic Effects

To determine if significant differences in larval growth rates (change in shell length and proportion tissue weight) and larval shell lengths were observed between treatments over the course of the experiment, a one-way analysis of variance (ANOVA) followed by a Tukey’s Honest Significant Difference (HSD) test was performed for each time point independently. One-way ANOVAs were also used to compare rates of metamorphosis across treatments, average juvenile shell lengths, and shell length growth rates between 1 and 4 DPM. Again, Tukey’s HSD tests were used to test for differences between levels within pH treatment. Assumptions for all parametric models (normality and equal residuals) were assessed via diagnostic plots. All data visualization and analyses were implemented in R v. 3.5.1 (R Core Team, 2018).

### RNA Isolation and Sequencing Preparation

In samples from Experiment I, RNA was isolated from a pool of approximately 15 individuals per replicate jar (*n* = 60 individuals/treatment) for larval gene expression (4- and 8-day) and approximately 4–20 individuals per replicate jar (*n* = 16–80 individuals/treatment) for juveniles (1 and 4 DPM). Total RNA was extracted from all samples using RNAqueous Total RNA Isolation Kit (Invitrogen, Waltham, MA, United States) per manufacturer’s instructions with the following modification: 0.5 mm glass beads (Sigma-Aldrich, St. Louis, MI, United States Z250465) were added to lysis buffer and samples were homogenized using a bead beater as per Kriefall et al. (2018). Trace DNA contamination was eliminated using *DNase1* (Invitrogen, Waltham, MA, United States AM2222) and gel electrophoresis confirmed RNA integrity and the absence of trace DNA. Approximately 500 ng of DNased total RNA was used to prepare tag-based RNAseq libraries, excluding samples that failed to yield at least 10 ng of DNased total RNA (*n* = 13; 9 larval, 4 juvenile). Libraries were prepared following Meyer et al. (2011) with appropriate modifications for Illumina Hi-Seq sequencing (Dixon et al., 2015; Lohman et al., 2016). Prepared libraries (*n* = 35) were sequenced across two lanes of Illumina Hi-Seq 2500 (50 bp single-end dual-indexed reads) at the Tufts University Core Facility (TUCF). RNA was similarly isolated from larvae in Experiment II. Replicates constituted a pool of approximately 25 individuals per jar (*n* = 100 individuals/treatment), and libraries (*n* = 36) were prepared, as described previously, from 500 ng DNased RNA and sequenced separately from Experiment I across two Illumina Hi-Seq 2500 (50 bp single-end dual-indexed reads) lanes.

### Transcriptome Assembly and Read Mapping

Our group had previously had success (i.e., Kriefall et al., 2018) mapping to the publicly available *Crepidula fornicata* transcriptome that was assembled using 454 sequencing data (Henry et al., 2010). Unfortunately, our libraries for these two experiments that were produced using the same TagSeq protocols used in Kriefall et al. (2018) resulted in poor mapping efficiencies to the previously published transcriptome. We therefore opted to assemble a novel *C. fornicata* transcriptome using all sequencing data from the current study. Data from all libraries were pooled, which yielded 533.8 million single-end reads. *Fastx_toolkit* trimmed *Illumina TruSeq* adapters and poly(A)+ tails, and reads were then quality filtered using *fastq_quality_filter* with the requirement that ≥80% of bases met a cutoff quality score of at least 20. These trimmed reads then served as input for RNAseq *de novo* assembly using Trinity (Grabherr et al., 2011) at the Shared Computing Cluster (SCC) at Boston University (BU). Ribosomal RNA contamination was identified as sequences exhibiting significant nucleotide similarity (BLASTN, *e*-value ≤ 1 × 10-8) to the SILVA LSU and SSU rRNAdatabases^1^ and these contigs were removed. Any contig >200 bp in length were annotated by BLAST sequence homology searches against UniProt and Swiss-Prot NCBI NR protein databases with an *e*-value cutoff of e^–5^ and annotated sequences were subsequently assigned to Gene Ontology (GO) categories (The UniProt Consortium, 2015). The transcriptome and its associated annotation files can be accessed at http://sites.bu.edu/davieslab/files/2019/05/Crepidula_fornicata _transcriptome_2019.zip.

As with the transcriptome, *fastx_toolkit* was used to remove *Illumina TruSeq* adapters and poly(A)^+^ tails from each individual sequence library. Short sequences (<20 bp) and low-quality sequences (quality score < 20) were also trimmed. A customized Perl script was used to remove PCR duplicates sharing the same degenerate header and transcript sequence^2^. Resulting quality-filtered reads were then mapped to the newly assembled *C. fornicata* transcriptome using B*owtie2.2.0* (Langmead and Salzberg, 2012) and a per-sample counts file was created using a custom Perl script. The script summed up reads for all genes and discarded reads that mapped to multiple genes. Mapped reads for Experiment I ranged from 199,558 to 1,557,855 per sample with mapping efficiencies ranging from 40.5 to 46.5% per sample (Supplementary Table 3), and reads for Experiment II ranged from 94,004 to 1,235,041 per sample with mapping efficiencies ranging from 40.7 to 46.2% per sample (Supplementary Table 4). It should be noted that these values exclude samples with low read counts, which were removed during downstream outlier analyses (Experiment I: larvae 8 day 7.6D and juveniles 1 DPM 7.5C and 4 DPM 8.0A; Experiment II: larvae 4 h 8.0B, 4 h 8.0D, 10 h 7.5D, 24 h 7.5B, and 24 h 7.5D) and one other sample that was removed as an outlier (Experiment I: juvenile 1 DPM 7.6C), which are detailed below.

### Gene Expression Analyses

Raw count data were first tested for outliers using the package *arrayQualityMetrics* (Kauffmann et al., 2009), and any samples that failed two or more outlier detection methods and had relatively low read counts (<130,000 for Experiment I and <90,000 for Experiment II; *n* = 4 for Experiment I and *n* = 5 for Experiment II, Supplementary Tables 3, 4) were excluded from subsequent analyses. Differential gene expression analyses on raw count data with outliers removed were then performed with *DESeq2* v. 1.22.2 (Love et al., 2014) in R v. 3.5.1 (R Core Team, 2018). Differentially expressed genes (DEGs, FDR: 0.05) between the reduced pH treatments (pH 7.5 and 7.6 in Experiment I and pH 7.5 in Experiment II) relative to the control (pH 8.0) were identified at each independent time point (4- and 8- days and 1 and 4 DPM in Experiment I and 4-, 10-, 24-, and 48-h in Experiment II) using generalized linear models (design = ∼ treatment) and *p*-values for the significance of these contrasts were generated using Wald statistics. These *p*-values were then adjusted using the false discovery rate method (Benjamini and Hochberg, 1995).

Data from raw counts files with outliers removed were r-log normalized and these data were then used as input for Principal Component Analyses (PCA) using the *prcomp* command to characterize overall differences in global gene expression across treatments and time points. Overall significance of constraints (day/hour, treatment) for each models were assessed using PERMANOVAs with the *adonis* function within the package *vegan* (Oksanen et al., 2016) with 999 permutations. To investigate magnitude of gene expression responses in juvenile *C. fornicata*, we compared the mean absolute value of overall log-fold change across all genes in the different larval treatments (pH 7.6 and 7.5) relative to control conditions (pH 8.0) and across the two timepoints (1 and 4 DPM) using ANOVA followed by Tukey’s HSD tests. Lastly, we compared the effect sizes within each pH treatment over time to quantify the degree of transcriptome elasticity (i.e., how quickly gene expression returned to baseline).

Gene ontology (GO) enrichment analyses were performed using Mann-Whitney *U* tests on ranked *p*-values (GO-MWU, Voolstra et al., 2011). GO enrichment was used to group together sets of genes as categories, based on their function, under the overarching divisions of “cellular component” (CC), “biological process” (BP), and “molecular function” (MF). Enrichment analyses allowed for a general overview of which categories were being differentially regulated under the reduced pH conditions. Negative logged *p*-values, used as a continuous measure of significance, were ranked and significantly enriched GO categories were identified. Results were plotted as dendrograms with hierarchical clustering of GO categories based on shared genes. Fonts and text colors were used to distinguish significance and direction of enrichment (up or down) for the regulated categories relative to pH 8.0. Gene annotation information, GO designation, raw mapped data counts, and *DESeq2* and GO enrichment results for both experiments can be accessed as supplementary files at https://github.com/chrislreyes/Crepidula.

## Results

### Effects of Reduced pH on Larval Growth, Metamorphosis, and Juvenile Shell Growth

**FIGURE 2:**
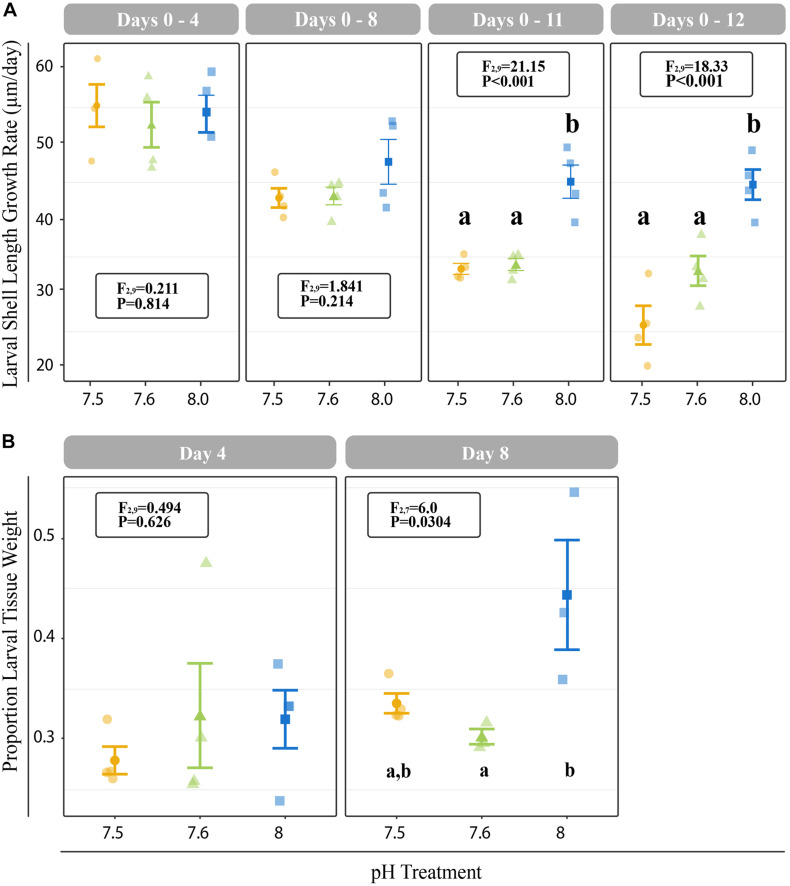
**(A)** Cumulative shell length growth rate (μm/day) for larvae relative to initial (day 0) measurements at 4-, 8-, 11-, and 12-days. **(B)** Proportion of tissue weight relative to total weight on days 4 and 8. Error bars represent +/- one standard error and different letters for pH treatments indicate significantly different means based on Tukey’s HSD tests (*P* < 0.05).

**FIGURE 3:**
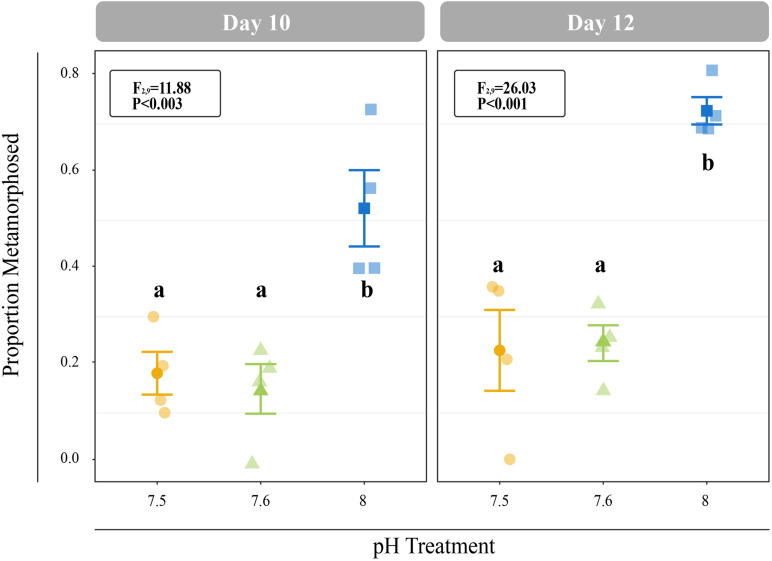
Proportion of larvae that metamorphosed after 6 h of induction with 20 mM elevated KCl at 10- and 11-days. Error bars represent +/- one standard error and different letters for pH treatments indicate significantly different means based on Tukey’s HSD tests (*P* < 0.05).

All *C. fornicata* larvae and juveniles from Experiment I were reared throughout the experiment with no mortality. Larvae reared at pH 7.5 and 7.6 grew significantly slower than those reared at pH 8.0 over the course of 12 days, although shell length growth rates were not significantly different for the first 8 days (Figure 2A). By day 11, larval shell lengths were at least 30% higher for larvae reared at control pH relative to those reared at either of the reduced pH levels (F_2,9_ = 21.15; *P* < 0.001; Figure 2A). While larval tissue weight as a proportion of total weight (tissue weight/total weight) did not differ significantly between treatments at day 4, larvae reared at pH 8.0 had a significantly higher tissue weight proportion by day 8 relative to larvae reared at pH 7.5 or 7.6 (F_2,7_ = 6.0; *P* = 0.0304; Figure 2B). Larval metamorphosis was significantly affected by rearing pH: a significantly smaller percentage of larvae reared at pH 7.5 and 7.6 metamorphosed during both 6-h induction periods (10- and 12-day) relative to larvae reared at pH 8.0 (*P* < 0.003; Figure 3).

**FIGURE 4:**
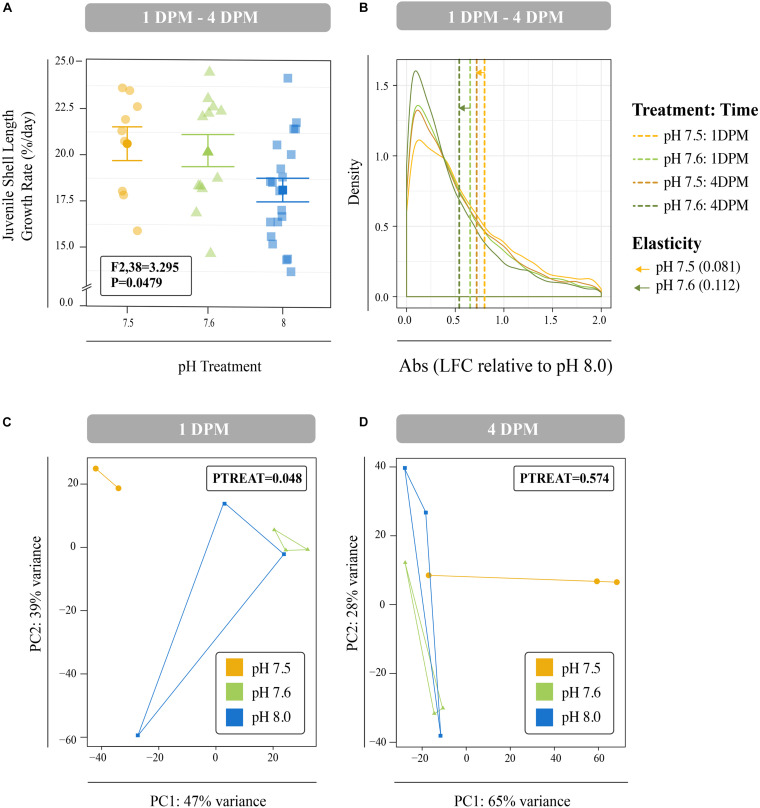
**(A)** Mean percent shell length growth rate (%/day) for juveniles between 1 DPM and 4 DPM that were raised in pH 7.5 and pH 7.6 as larvae and then placed in control conditions post settlement. Error bars represent +/- one standard error. Overall difference between pH treatments was significant (*P* = 0.049), however, no Tukey’s HSD test passed the 0.05 *p*-value cut-off suggesting no significant differences between any two levels of a factor. **(B)** Mean log-fold change (absolute values) across all isogroups in the different larval treatments (pH 7.6 and 7.5) relative to control conditions (pH 8.0) across the two timepoints (1 DPM and 4 DPM). Mean values are denoted with dashed lines and distances between means within each treatment represent transcriptome elasticity, which is our proxy for recovery. Principal coordinate analyses (PCA) of all r-log transformed isogroups in juveniles clustered by experimental treatment at 1 DPM **(C)** and 4 DPM **(D)**. Overall responses of gene expression across pH treatments were found to be significant at 1 DPM, but no longer at 4 DPM. Colors indicate pH treatment condition: blue = pH 8.0, green = pH 7.6, and yellow = pH 7.5.

Juveniles reared at reduced pH as larvae had significantly smaller shell lengths at both 1 DPM and 4 DPM in comparison to juveniles reared at ambient pH as larvae (*P* < 0.001; Supplementary Figures 1B,C). However, these reduced juvenile shell lengths were due to reduced larval shell lengths observed from day 8 onward (Supplementary Figure 1A) and significant differences in juvenile shell length growth rates were not observed after multiple test correction (*P* = 0.0479; Tukey’s HSD > 0.05; Figure 4A).

### Transcriptomic Responses of *Crepidula fornicata* to Reduced pH

**FIGURE 5:**
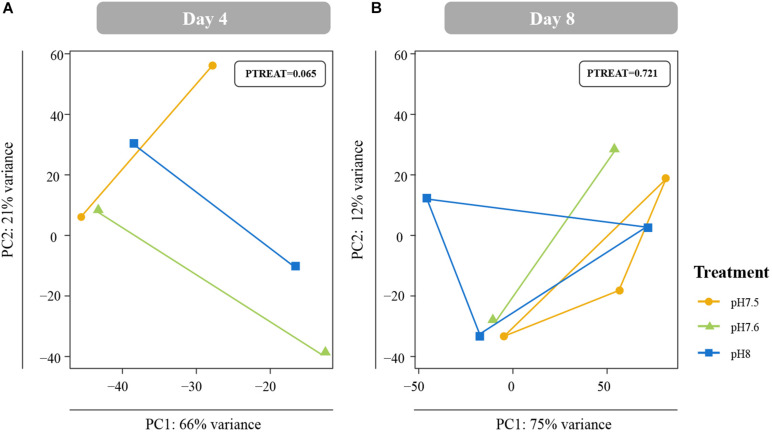
Principal coordinate analyses (PCA) of all r-log transformed isogroups in *Crepidula fornicata* larvae clustered by experimental treatment at 4 days **(A)** and 8 days **(B)** old. Overall responses of *C. fornicata* larval gene expression across pH treatments were found to be insignificant at both time points. Colors indicate pH treatment condition: blue = pH 8.0, green = pH 7.6, and yellow = pH 7.5.

Differences in global gene expression in *C. fornicata* larvae were better explained by larval age (4-day vs. 8-day) than by pH treatment (Supplementary Figure 2B, Figure 5), although neither of these factors resulted in significant sample clustering. In contrast, larval pH treatment had a significant influence on juvenile snail gene expression at 1 DPM (*p* = 0.048); however, these differences were no longer significant at 4 DPM (Figures 4C,D), suggesting *C. fornicata* acclimation. Juvenile age had no overall effect on gene expression (Supplementary Figure 2C). While there were trends in global gene expression differences between pH treatments, numbers of differentially expressed genes (DEGs) were generally very low, especially in larvae. Relative to larvae reared at pH 8.0, larvae reared at pH 7.5 only downregulated a single gene at 8-d and no DEGs were detected for larvae reared at pH 7.6 at 4- or 8-days. Juveniles that had been reared as larvae at pH 7.5 differentially expressed 92 genes (65 upregulated; 27 downregulated) at 1 DPM and 210 genes (140 upregulated; 70 downregulated) at 4 DPM in comparison to juveniles that had been reared as larvae at pH 8.0, and juveniles that had been reared as larvae at pH 7.6 differentially expressed 23 genes (2 upregulated; 21 downregulated) at 1 DPM but only a single gene (upregulated) at 4 DPM. However, when we explored the differences in log-fold change across the different pH treatments and time point, we found that juveniles that were reared in pH 7.5 exhibited larger gene expression differences than those reared at pH 7.6 and this was observed at both time points (1 DPM, 4 DPM; Figure 4B). Lastly, when we compared the effect sizes within a treatment across timepoints (our proxy for transcriptome elasticity), we found that elasticity of juveniles reared at pH 7.6 was greater (0.112) than the elasticity of the juveniles reared at pH 7.5 (0.081), suggesting that those reared at pH 7.6 recovered at a faster rate.

### GO Enrichment in Response to Reduced pH of *Crepidula fornicata* Larvae

**FIGURE 6:**
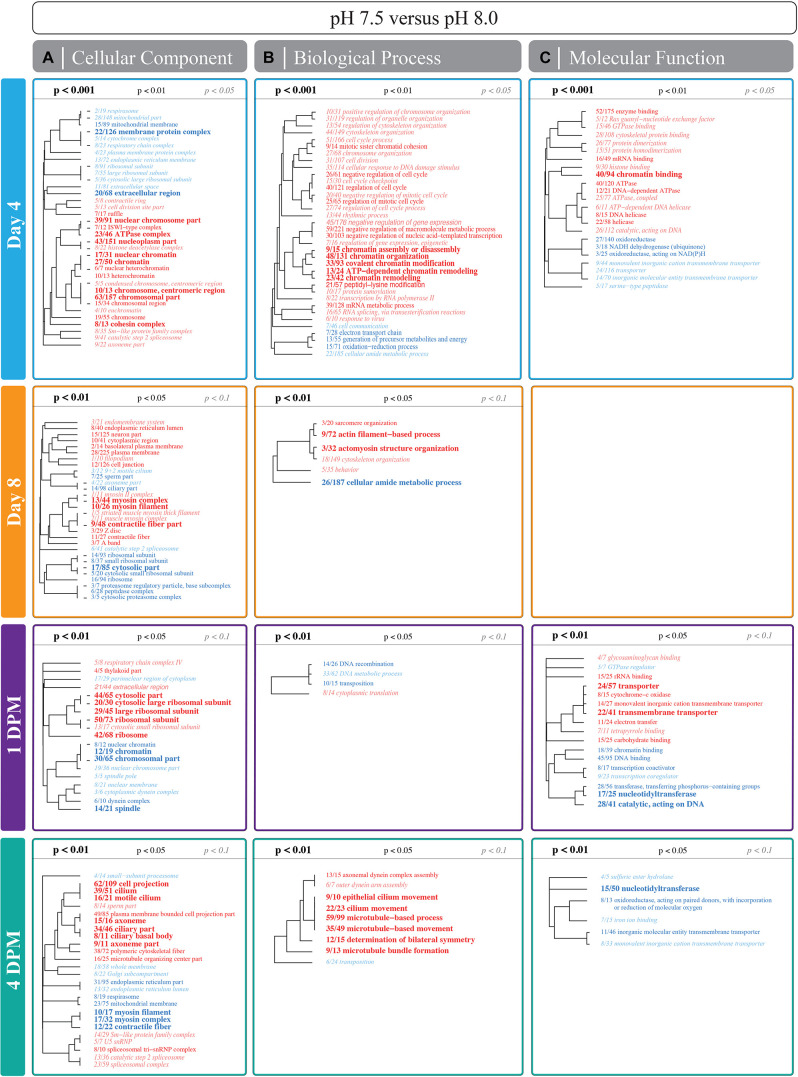
Significantly enriched gene ontology (GO) categories for the pairwise comparison between pH 7.5 and 8.0 treatments for larvae and juveniles in Experiment I. Mann-Whitney U (MWU) tests were conducted based on ranking of signed log *p*-values and results were plotted as dendograms with an indication of genes shared between categories. Enrichment by **(A–C)** “cellular component,” “biological process,” and “molecular function” (columns) are shown for 4- and 8-days for larvae and for 1 DPM and 4 DPM (rows) for juveniles. Overrepresented categories relative to pH 8.0 are colored as red and underrepresented categories are colored as blue. A blank grid indicates that there were no significantly enriched categories for that division at that time point. Results for pH 7.6 and 8.0 can be found in Supplementary Figure 3.

In *C. fornicata* larvae, most GO enrichments were observed to be overrepresented under reduced pH treatments relative to pH 8.0 (red text), and there were consistently more GO enrichments detected in 4 day old larvae relative to 8 day old larvae (Figure 6 and Supplementary Figure 3). GO categories associated with ribosomal proteins (i.e., *ribosomal subunit*; GO:00443912, *small ribosomal subunit*; GO:00159354, *structural constituent of the ribosome*; GO:00037352) were consistently downregulated at 4- and 8-days in larvae reared under pH 7.5 in comparison to larvae reared at pH 8.0 (Figure 6) and in 4-days larvae reared under pH 7.6 (Supplementary Figure 3). GO categories associated with mitochondrial proteins (i.e., *mitochondrial part*; GO:00444292, *respirasome*; GO:00704692, and *mitochondrial membrane*; GO:0005743) were also found to be downregulated in 4-d larvae reared under pH 7.5 and in 4- and 8-days larvae reared under pH 7.6. In contrast, terms associated with cytoskeleton proteins (i.e., *cytoskeleton*; GO:00058562, *cytoskeleton organization*; GO:00070102, and *regulation of cytoskeleton organization*; GO:00514932) were consistently upregulated in 4- and 8-days larvae reared under pH 7.5 and in 4-days larvae reared under pH 7.6. Additionally, GO categories associated with oxidative stress responses (i.e., *oxidoreductase activity*; GO:00164912, *oxidoreductase, acting on NAD(P)H*; GO:00166512, and *oxidation-reduction process*; GO:00551142) were downregulated in 4-d larvae reared under pH 7.5 and in 4- and 8-d larvae reared under pH 7.6.

### *Crepidula fornicata* Juvenile GO Enrichment in Response to Larval Rearing at Reduced pH

In contrast to larval responses, most GO enrichments detected for juveniles derived from larvae reared at pH 7.6 were found to be largely underrepresented (blue text; Supplementary Figure 3) while those derived from larvae reared at pH 7.5 were under- and overrepresented (Figure 6). Juveniles derived from larvae from both reduced pH treatments showed enrichment of ribosomal protein GO categories at 1 DPM, whereas those derived from larvae reared at pH 7.6 exhibited the opposite pattern in those same categories at 4 DPM (Figure 6 and Supplementary Figure 3). Similarly, juveniles derived from larvae reared at pH 7.6 downregulated categories associated with the cytoskeleton at 1 DPM (i.e., *polymeric cytoskeletal fiber*; GO:0005874, *microtubule-based process*; GO:00070172, and *microtubule-based movement*; GO:00070182), yet juveniles derived from larvae reared at pH 7.5 exhibited enrichment of those same categories at 4 DPM (Figure 6 and Supplementary Figure 3). In contrast to larvae, juvenile *C. fornicata* derived from larvae reared at pH 7.5 upregulated mitochondrial proteins at 1 DPM. Juveniles derived from larvae reared at pH 7.6, however, downregulated these same GO terms at 4 DPM, again demonstrating the more dynamic responses of juveniles. Lastly, GO categories associated with oxidative stress responses were not differentially enriched at 1 DPM, but were downregulated at 4 DPM in juveniles derived from larvae reared at pH 7.5 only.

### 48-h Reduced pH Experiment: Short Term Larval Transcriptomic Responses and GO Enrichment

As observed for larvae in Experiment I, differences in global gene expression for larvae in Experiment II were better explained by larval age than by pH treatment (Supplementary Figure 2A). Relative to pH 8.0, larvae reared at pH 7.5 differentially expressed 2 genes (1 upregulated; 1 downregulated) at 4 h and 33 genes (18 upregulated; 15 downregulated) at 48 h (FDR-adjusted <0.1), while no genes were differentially expressed at 10 or 24 h. In larvae reared under pH 7.5, GO categories associated with ribosomal proteins were underrepresented at 4 h, overrepresented at 10 and 24 h and then underrepresented again at 48 h relative to larvae reared at pH 8.0 (Supplementary Figure 4). GO categories associated with mitochondrial proteins were overrepresented at 4 and 10 h and then underrepresented at 48 h in larvae reared under pH 7.5. Lastly, categories associated with oxidative stress responses were overrepresented at 10 h but these same categories were underrepresented at 48 h. Overall these responses suggest that larval *C. fornicata* exhibit initial transcriptomic responses to reduced pH, but these responses are reversed after 48 h in treatment, demonstrating the transcriptome plasticity of larval *C. fornicata* in response to acute reduced pH.

## Discussion

### Low pH Reduced Larval Growth in *Crepidula fornicata*

Consistent with other studies investigating the effects of reduced pH on larval growth rates in *C. fornicata* (Kriefall et al., 2018; Bogan et al., 2019), we observed reduced shell and tissue growth rates for larvae reared at reduced pH. These reductions were not immediately apparent and only became evident after 8 days of exposure for tissue growth and after 11 days of exposure for shell length increases (Figure 2). These results are in line with previous studies on *C. fornicata*’s congener *Crepidula onyx*, which also did not exhibit immediate growth rate reductions and slower larval growth was only observed after 14 days of rearing at reduced pH (7.3 and 7.7; Maboloc and Chan, 2017). Reduced growth rates in response to lower pH levels have also been observed across diverse taxa, including coral and algae, whose growth were also negatively affected after 2 weeks of rearing at various elevated *p*CO_2_ treatments (55, 70, 100, and 210 Pa; Comeau et al., 2014). In contrast, no changes in shell length or development were found for embryos of the Olympia oyster *Ostrea lurida* after exposure to pH conditions as low as 7.4 (Waldbusser et al., 2016). It is worth noting, however, that these oyster embryos were only reared at reduced pH conditions for 5 days, so perhaps chronic conditions may have reduced growth. In our study, 8 days of continuous exposure to reduced pH was required before *C. fornicata* larvae exhibited detectable phenotypic responses. Clearly, the amount of time an organism spends under experimental treatment is a particularly important consideration when assessing an organism’s response to OA and these responses are likely to vary for organisms with different life history characteristics.

It has been previously suggested that reducing investment in calcification may minimize the energetic burden of living in reduced pH environments and serve as a potential mechanism of acclimation and resilience (Waldbusser et al., 2016). Indeed, the potential to acclimate to OA and maintain calcification rates has been proposed to be energetically costly (i.e., Cohen and McConnaughey, 2003). Previous work on larval sea urchins exposed to reduced pH found reduced growth coupled with increased metabolic rates (Stumpp et al., 2011). Davies et al. (2016) also demonstrated that reduced calcification rates in adult corals were coupled with increased expression of genes associated with metabolism. Similarly, Comeau et al. (2014) noted that rapidly calcifying corals were more sensitive to the impacts of OA than slow calcifiers. While we did not explore metabolic rates of *C. fornicata*, we propose that larval *C. fornicata* may alter their growth after experiencing prolonged reduced pH in order to minimize their energetic burden, which may explain the exceptional resilience of this species in response to a variety of stressors (Diederich and Pechenik, 2013; Noisette et al., 2016; Kriefall et al., 2018). Regardless, future work should explore these types of research questions across additional *C. fornicata* broods to confirm that the responses we observed here are conserved across genetic backgrounds.

### Transcriptomic Effects Preceded Detectable Phenotypic Effects in *Crepidula fornicata* Larvae

Gene expression patterns of *C. fornicata* larvae under OA after 4- and 8-d suggested downregulation of genes associated with growth and metabolism, and these patterns preceded reductions in larval shell length that became evident at 11- and 12-days (Figure 2A). At 4-days, we observed downregulation of genes associated with mitochondria, ribosomal structures, and electron transport (Figure 6 and Supplementary Figure 3), which are pathways consistent with reduced growth and reduced respiration (De Wit et al., 2016). We also observed downregulation of genes associated with oxidation-reduction processes (Figure 6 and Supplementary Figure 3), which, in conjunction with downregulation of electron transport, may further reflect metabolic demands associated with OA. At 8-days, larval gene expression changes were not as pronounced, but reductions in ribosomal categories associated with growth were maintained. Despite these GO enrichments at 4- and 8-days, a significant overall transcriptomic effect of pH treatment was not detected (Figure 5). In addition, the changes in expression of genes associated with growth at 4- and 8-days did not manifest phenotypically until 11-days (Figure 2). Indeed, major gene expression changes have previously been found to precede phenotypic effects in corals under reduced pH (7.6–7.7) over the course of a 28-day experiment (Kaniewska et al., 2012). Overall, our results suggest that the expression of genes associated with metabolism and growth is influenced by OA and that these patterns of gene expression may be powerful predictors of downstream phenotypic changes.

### Rearing at Reduced pH Delayed Competence for Metamorphosis

On days 11 and 12, lower proportions of larvae reared at pH 7.5 and 7.6 metamorphosed within 6 h of induction relative to larvae reared at control conditions (Figure 3), suggesting that OA delays the onset of metamorphic competence in *C. fornicata*. This result recapitulates findings from Kriefall et al. (2018) and Bogan et al. (2019), which found that larval *C. fornicata* exposed to reduced pH conditions took a significantly longer time to become competent to metamorphose. Delayed onset of metamorphic competence in response to reduced pH has been observed in a wide array of calcifying marine invertebrates, including *Mercenaria mercenaria* (hard clams), *Argopecten irradians* (bay scallops), and *Crassostrea virginica* (Eastern oysters) (Talmage and Gobler, 2009). More importantly, however, competent *C. fornicata* larvae exposed to reduced pH (7.5) were not inhibited from metamorphosing in response to a natural adult-derived cue (Pechenik et al., 2019). Thus, while reduced pH conditions may delay the onset of competence to metamorphose, they do not inhibit already competent larvae from undergoing metamorphosis, further exemplifying *C. fornicata’*s resilience to OA. Given that reduced pH delays the onset of competence, future work might assess whether reduced pH also impacts the length of time required to respond to cues for metamorphosis. Lengthening the time required for a response, like delaying the onset of competence to metamorphose, could increase the potential for predation by lengthening time spent in the water column and increasing risk of mortality (Ross et al., 2016). This additional time in the pelagic environment may also influence a species’ dispersal capacity and potentially lead to range expansion and invasion events, which have been widely observed in *C. fornicata* (Blanchard, 2009; Bohn et al., 2012).

### *Crepidula fornicata* Exhibited no Carry-Over Effects on Juvenile Growth From Reduced pH Experienced as Larvae

The effects of OA have been shown to frequently transcend life history stages, with exposure at earlier stages influencing development and physiology at later stages (Hettinger et al., 2013; Ross et al., 2016; Bogan et al., 2019). Here, we observed smaller shell lengths for 1 DPM and 4 DPM juveniles that were reared as larvae under reduced pH (Supplementary Figures 1B,C). However, these reduced shell lengths were a direct consequence of reduced growth observed in larvae at 11- and 12-days (Supplementary Figure 1A) and no significant difference in rates of juvenile shell growth were observed, despite differences in larval rearing conditions (Figure 4A). Thus, in this experiment, larval pH treatment did not result in carry-over effects on growth for juveniles of *C. fornicata* that had been transferred to control conditions following metamorphosis. This result contrasts with those of Bogan et al. (2019), who found negative effects of similar larval pH treatments on the post-metamorphic growth of juvenile *C. fornicata* in three experiments conducted on two different populations across two different seasons. Similarly, Pechenik and Tyrell (2015) found variation between broods in the carry-over effects in juvenile growth resulting from larval nutrition quality. Despite such evidence for intraspecific variation in carry-over effects in *C. fornicata*, other studies investigating carry-over effects on growth in other molluscs have shown that these effects can persist for months post settlement. For example, Gobler and Talmage (2013) showed that juvenile *Argopecten irradians* (bay scallops) reared at elevated *p*CO_2_ (750 μatm) as larvae experienced reduced growth relative to juveniles reared at ambient *p*CO_2_ (390 μatm) as larvae and these carry-over effects did not dissipate for 10 months. Given the intraspecific variation in the expression of carry-over effects in *C. fornicata*, future studies should assess carry-over effects across multiple broods and profile the transcriptome of broods expressing carry-over effects and compare them to those of broods not expressing these effects in response to these same conditions.

### Transcriptome Elasticity in Juvenile *Crepidula fornicata* Facilitated Recovery From Low pH Treatment Experienced as Larvae

Analyses of juvenile transcriptomic data at 1 and 4 DPM suggest that snails exposed to reduced pH as larvae maintain signatures of these OA impacts as juveniles, however, these impacts dissipate over time and recovery (i.e., transcriptome elasticity) is slower if more severe OA conditions were experienced (Figures 4B–D). The effect of OA treatment on overall gene expression patterns of juveniles at 1 DPM was significant (Figure 4C) and these patterns were associated with the enrichment of genes associated with mitochondria, ribosomal structures, and electron transport, which suggest increased growth, respiration, and metabolism (Figure 6, Supplementary Figure 3). These patterns are consistent with carryover effects from conditions experienced as larvae (i.e., juveniles are having to work harder to compensate for prior conditions). It has been suggested that some organisms have the ability to increase rates of their biological processes, including metabolism and growth, to compensate for increased seawater acidity (Wood et al., 2008; Gutowska et al., 2010; Lannig et al., 2010). This adaptation, however, comes at a cost and was found to be coupled with reductions in muscle mass in brittlestars (Wood et al., 2008) and a reduction in the incorporation of chitin in the cuttlebones of cephalopods (Gutowska et al., 2010). While we did not observe reduced growth rates in juvenile *C. fornicata* here, it is possible that increased growth and metabolism suggested by the gene expression profiles of juvenile *C. fornicata* similarly involved a phenotypic trade-off that was not quantified in the current study.

In contrast to juveniles at 1 DPM, juveniles at 4 DPM exhibited downregulation of genes associated with mitochondria, ribosomal structures, and oxidation-reduction processes, suggesting reduced energetic output. After upregulating genes at 1 DPM in response to pH conditions experienced as larvae, juveniles may downregulate these core pathways at 4 DPM in the absence of pH treatment in order to return to baseline expression levels, which is consistent with the reduced mean log-fold change in 4 DPM juveniles relative for 1 DPM juveniles for both pH treatments. Upregulation of genes associated with ciliary structures at 4 DPM suggests that juveniles may be increasing feeding activity upon recovery from pH conditions. Feeding rate is an important determinant of growth and survival; therefore, it would be an informative phenotype to quantify in future OA studies on *C. fornicata*. Overall, our findings suggest that within a period of 4 days, juvenile *C. fornicata* show evidence of transcriptome recovery (i.e., elasticity) from OA conditions experienced as larvae through acclimation and gene expression plasticity.

### *Crepidula fornicata* Larvae Exhibited Transcriptomic Resilience in Response to Acute pH Treatment

Gene expression changes in response to environmental change can occur quickly (Evans and Hofmann, 2012) and monitoring these changes through time can allow for a more in-depth characterization of how stressors influence an organism across short time scales. To test the effects of acute reduced pH conditions, we assessed gene expression responses of *C. fornicata* larvae reared at reduced pH (7.5) relative to larvae reared at higher pH (8.0) over the course of 48 h. Larval transcriptomes responded rapidly to OA within the first 24-h, upregulating genes associated with mitochondria, ribosomal structures, and oxidation-reduction, suggestive of increased respiration, growth, and metabolism (Supplementary Figure 4). Regulation of these gene ontology terms have been consistently observed in the literature (reviewed in Strader et al., 2020). For example, Pan et al. (2015) found that *Strongylocentrotus purpuratus* urchin larvae under acidification (∼800 μatm *p*CO_2_) increased protein synthesis by approximately 50%. They determined that the majority of available ATP (84%) was accounted for in protein synthesis and ion transport alone, which is indicative of metabolic stress as most energy is directed toward making proteins and offsetting the effects of OA. Basal metabolism was similarly found to increase in the bivalve *Laternula elliptica* after exposure to reduced pH conditions (pH 7.78) over the course of 120 days (Cummings et al., 2011). Our results suggest that increases in expression of genes associated with metabolism and protein synthesis in *C. fornicata* larvae begin to occur at the onset of exposure to reduced pH and that this rapid response may play a role in the ability of the species to acclimate quickly to OA.

Indeed, we observed reduced overall transcriptomic responses and downregulation of the same terms that were upregulated within the first 24-h by 48-h, which indicates that larvae were able to acclimate and recover from acute OA treatment (Supplementary Figure 4). Although physiological measurements were not taken for these larvae, if reared for a longer duration, it is likely that larvae reared in reduced pH (7.5) would have exhibited reduced growth, consistent with Experiment I (Figure 2). Given that no previous study has assessed the transcriptomic effects of OA at hourly scales, these findings highlight the importance of the ephemeral transcriptional changes involved in acclimation to OA that may be overlooked in longer term experiments with coarser sampling resolution.

### *Crepidula fornicata* Exhibit Resilience to Reduced pH Conditions Through Transcriptome Plasticity

Overall, our findings suggest that *C. fornicata* exhibit dynamic phenotypic and transcriptomic responses across both larval and juvenile life history stages in response to OA consistent with gene expression plasticity (Rivera et al., 2021). At the transcriptome level, larval gene expression patterns were suggestive of stress when the larvae were initially reared in reduced pH treatments (10-h), but these patterns were no longer detectable by 48-h (Supplementary Figure 4). Similarly, juveniles that were reared under OA conditions as larvae exhibited gene expression patterns consistent with stress at 1 DPM (Figure 6, Supplementary Figure 3). However, these carry-over effects from the larval stage dissipated by 4 DPM through transcriptome elasticity (Figure 4B). The ability of *C. fornicata* larvae and juveniles to acclimate to OA quickly sheds light onto the mechanisms underlying this species’ resilience, which likely results from its life spent in variable pH intertidal environments. Reduced larval growth at 11- and 12-days, which appears to be preceded by transcriptomic effects at 4- and 8-days, may be consistent with OA acclimation in *C. fornicata*. Reduced growth and metabolism may lessen the energetic burden under OA exposure and may provide clues into how these snails are able to exhibit such broad resilience. However, these reductions in shell growth may also be independent of biological control and simply result from the reduced efficacy of calcification in undersaturated seawater (Kapsenberg et al., 2018). Overall, our gene expression results in *C. fornicata* corroborate the findings of a recent review by Strader et al. (2020), which observed metabolic processes, calcification, and stress responses consistently regulated by marine metazoans in response to OA. However, we also acknowledge that while changes in gene expression patterns in this study tended to correlate with changes in phenotypes, previous work in oysters (*Crassostrea gigas*) has observed that some phenotypes (e.g., the rates of ion transport) could not be predicted from concurrent measurements of gene expression or enzyme activity (Pan et al., 2016). These sorts of findings suggest that future work should continue to include multiple sampling times of both gene expression and phenotype, which may lead to stronger links between genotype and phenotype, given that gene expression changes likely precede the phenotypic changes observed here. Also, the experiments conducted here were relatively acute stress treatments and *C. fornicata* responses are likely to be modulated by more chronic exposures. In addition, each of these experiments was conducted on only a single brood of *C. fornicata* and the genetic variation in these responses remains unexplored. Future work should incorporate larvae from more diverse genetic backgrounds to explore the extent of this variation. Lastly, in order to fully characterize the dynamic effects of OA on *C. fornicata*, further studies are needed to discern the influence of multiple stressors along with daily and seasonal environmental fluctuations across life history stages (e.g., Waldbusser and Salisbury, 2014; Rivest et al., 2017). Given that differences in growth and metabolic rates have been found for organisms exposed to static versus fluctuating pH treatments (Cornwall et al., 2013; Roleda et al., 2015; Britton et al., 2016; Mangan et al., 2017), we suggest that future work should examine the phenotypic and transcriptomic responses across different treatment durations and variable/static OA conditions.

## Data Availability Statement

The raw sequence data files are available in the NCBI Sequence Read Archive (SRA) under project number PRJNA549522 and accession numbers SRX6090329–SRX6090399. Assembled transcriptome and its associated annotation files can be accessed at https://sites.bu.edu/davieslab/data-code. All scripts, raw mapped data counts, and DESeq2 and GO enrichment results for both experiments can be accessed as supplementary files at https://github.com/chrislreyes/Crepidula.

## Author Contributions

AP, JP, and SD designed the experiments. ML, AP, and JP conducted the experiments. BB and CR-G completed all molecular work and TagSeq library preparations. CR-G and XC performed all statistical and bioinformatic analyses with supervision from SD. CR and SD drafted the manuscript. All authors edited and approved the manuscript.

## Conflict of Interest

The authors declare that the research was conducted in the absence of any commercial or financial relationships that could be construed as a potential conflict of interest.

## Publisher’s Note

All claims expressed in this article are solely those of the authors and do not necessarily represent those of their affiliated organizations, or those of the publisher, the editors and the reviewers. Any product that may be evaluated in this article, or claim that may be made by its manufacturer, is not guaranteed or endorsed by the publisher.

## Footnotes

1
http://www.arb-silva.de/

2
https://github.com/z0on/tag-based_RNAseq
