# Supplementary material for: The Marine Gastropod Crepidula fornicata Remains Resilient to Ocean Acidification Across Two Life History Stages
Source: Front Physiol. 2021 Aug 25;12:702864. doi: 10.3389/fphys.2021.702864 (PMC8424201; doi:10.3389/fphys.2021.702864)

**Supporting Information:**

**The marine gastropod *Crepidula fornicata* remains resilient to ocean acidification across two life history stages**

Christopher L Reyes^1^, Brooke E Benson^1^, Morgan Levy^2^, Xuqing Chen^1^, Anthony Pires^3^, Jan A Pechenik^2^ and Sarah W Davies^1*^

^1^Biology Department, Boston University, Boston, MA, USA

^2^Biology Department, Tufts University, Medford, MA USA

^3^Biology Department, Dickinson College, Carlisle, PA

Figure S1: A. Mean larval shell length (µm) in each pH treatment measured at 4, 8, 11, and 12 days in treatment. Mean juvenile shell length growth rates (µm/day) measured 24 hours post settlement (B. 1-DPM) and 4 days later (C. 4-DPM). Error bars represent +/- one standard error and different letters for pH treatments indicate significantly different means based on Tukey’s HSD tests (*P* < 0.05).


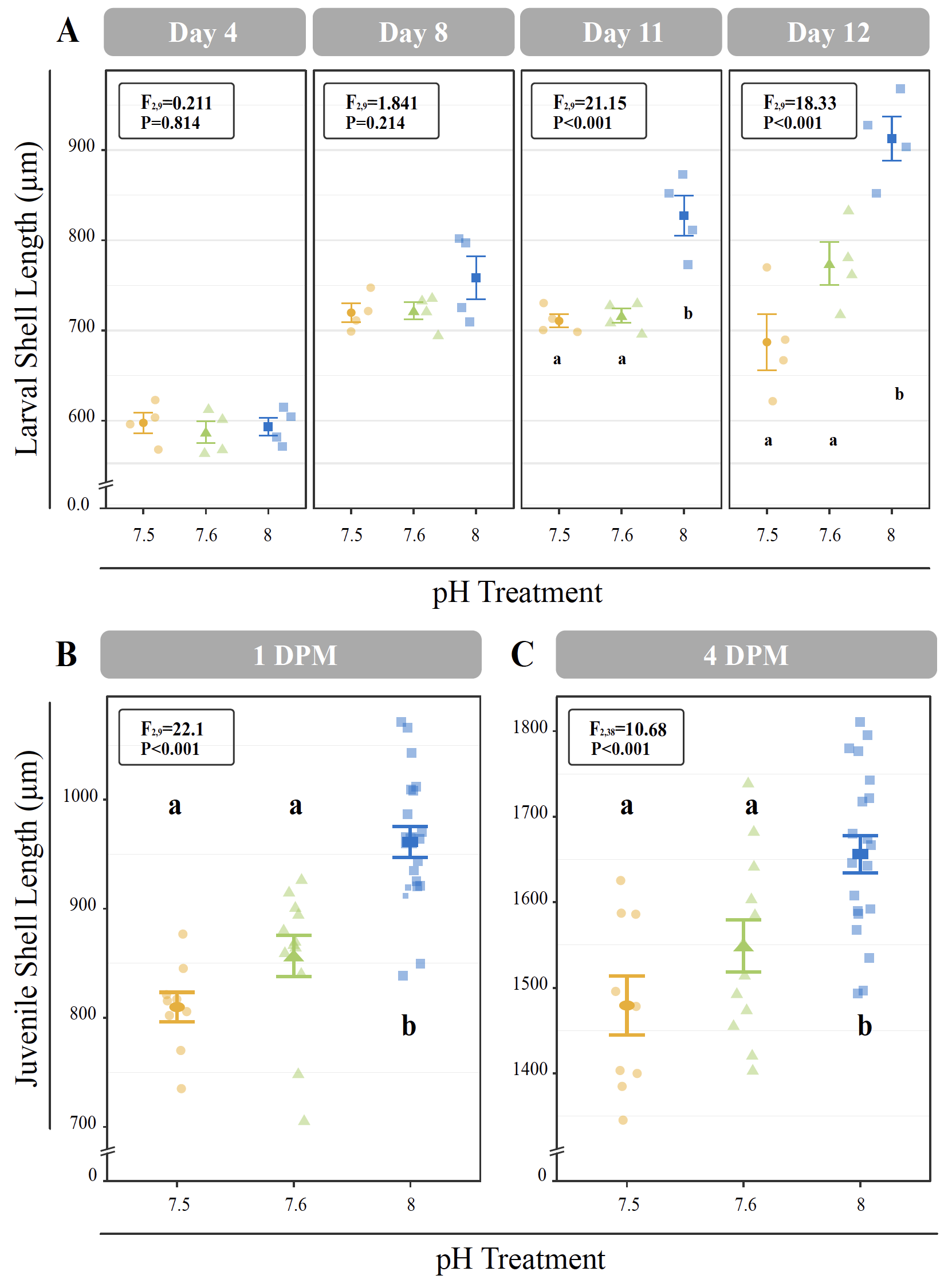


Figure S2: Principal coordinate analyses (PCA) of all r-log transformed isogroups in Experiment II (A) and Experiment I larvae (B) and Experiment II juveniles (C) with each sample colored by treatment and samples clustered by time points. Overall responses of gene expression across pH treatments were found to be insignificant in experiment I, insignificant for larvae from Experiment I, and significant for juveniles of Experiment I. Colors indicate pH treatment condition: blue = pH 8.0, green = pH 7.6, yellow = pH 7.5. Time point was insignificant for Experiment II, significant across larvae in Experiment I, and insignificant in juveniles in Experiment I.


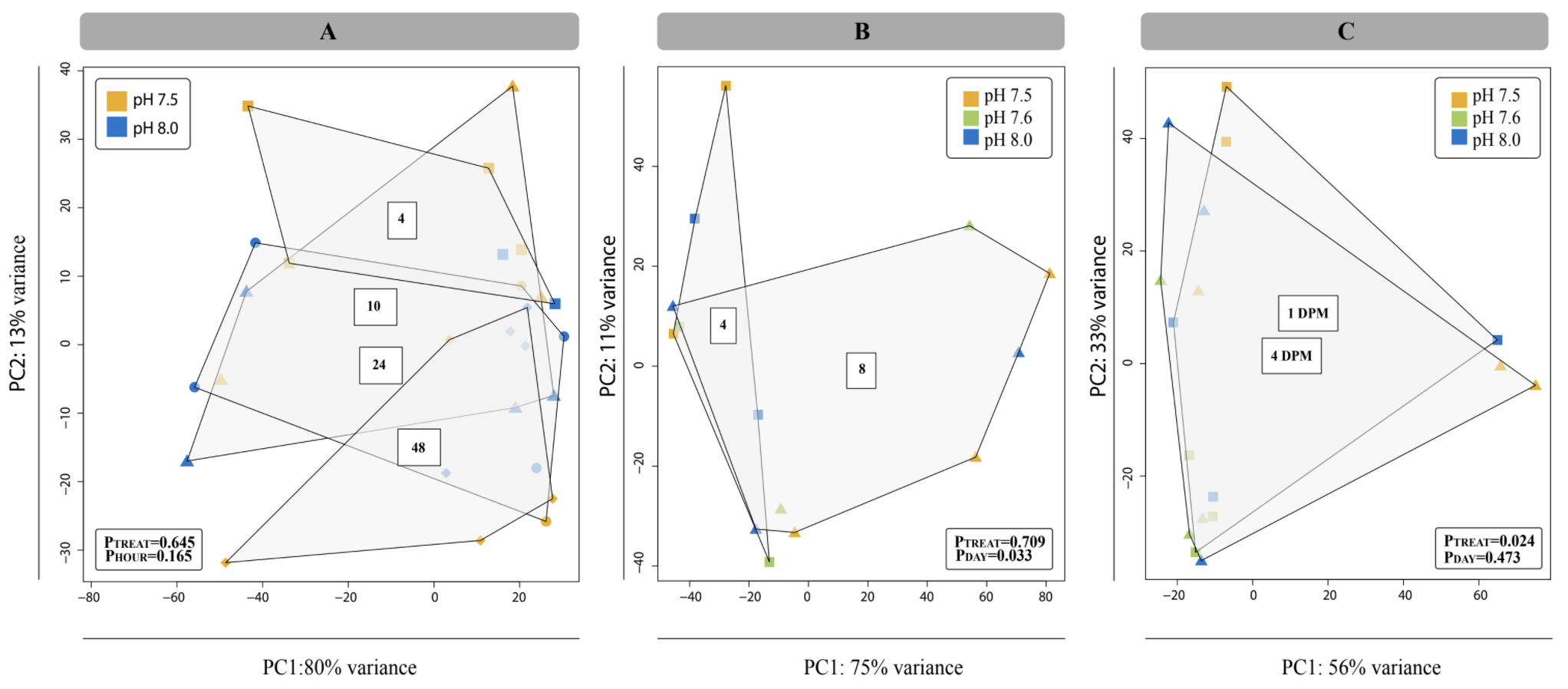


Figure S3: Significantly enriched gene ontology (GO) categories for the pairwise comparison between pH 7.6 and pH 8.0 treatments for larvae and juveniles in Experiment I. Mann-Whitney U (MWU) tests were conducted based on ranking of signed log p-values and results were plotted as dendograms with an indication of genes shared between categories. Enrichment by 'cellular component', 'biological process', and 'molecular function' (columns) are shown for 4- and 8-d for larvae and for 1 DPM and 4 DPM (rows) for juveniles. Overrepresented categories relative to pH 8.0 are colored as red and underrepresented categories are colored as blue. A blank grid indicates that there were no significantly enriched categories for that division at that time point. Results for pH 7.5 and 8.0 can be found in Figure 6.


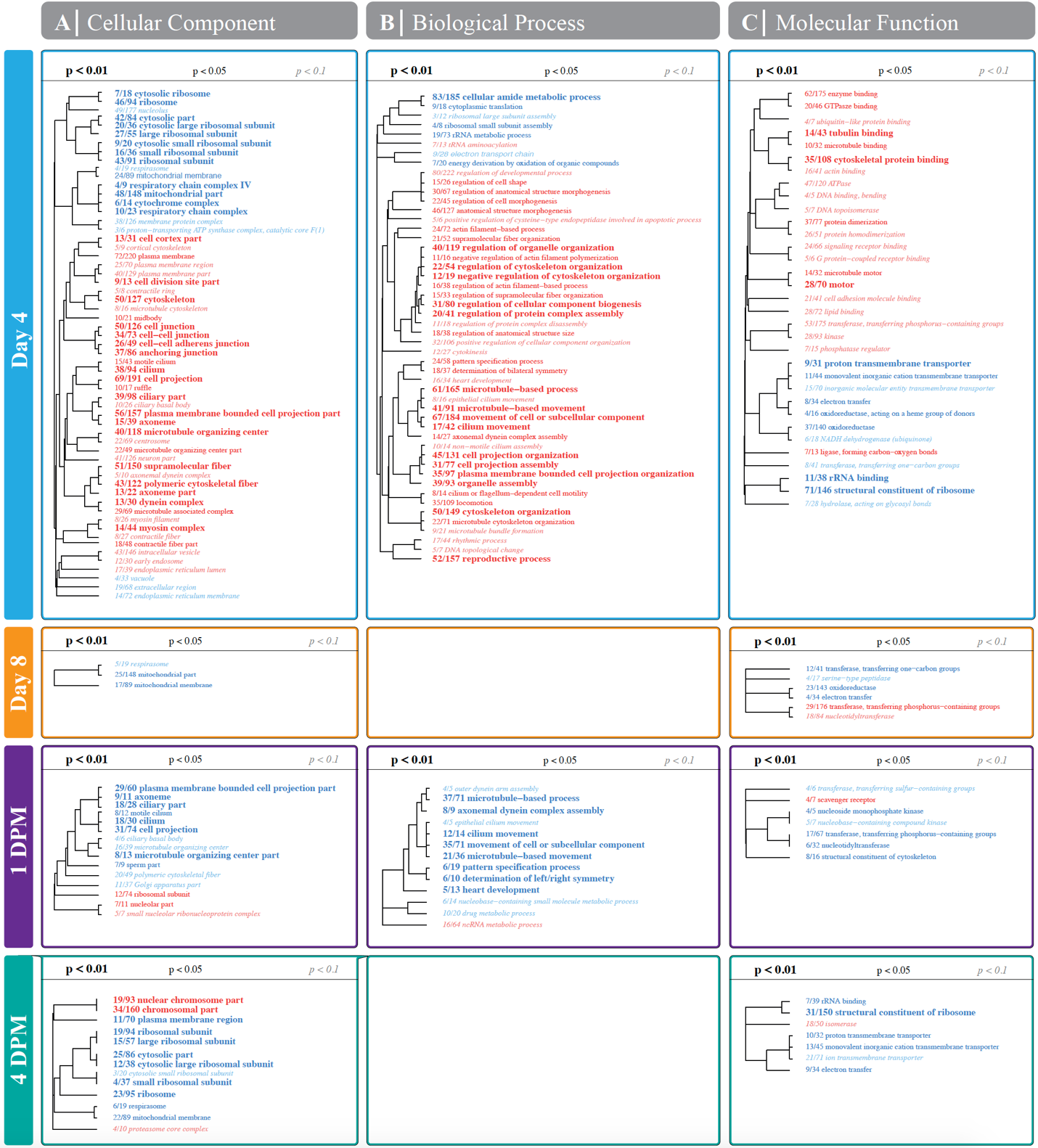


Figure S4: Significantly enriched gene ontology (GO) categories for the pairwise comparison between pH 7.5 and pH 8.0 treatments for Experiment II larvae. Mann-Whitney U (MWU) tests were conducted based on ranking of signed log p-values and results were plotted as dendograms with an indication of genes shared between categories. Enrichment by 'cellular component', 'biological process', and 'molecular function' are shown for 4-, 10, 24, and 48-hr larvae. Overrepresented categories relative to pH 8.0 are colored as red and underrepresented categories are colored as blue. A blank grid indicates that there were no significantly enriched categories for that division at that time point.

**
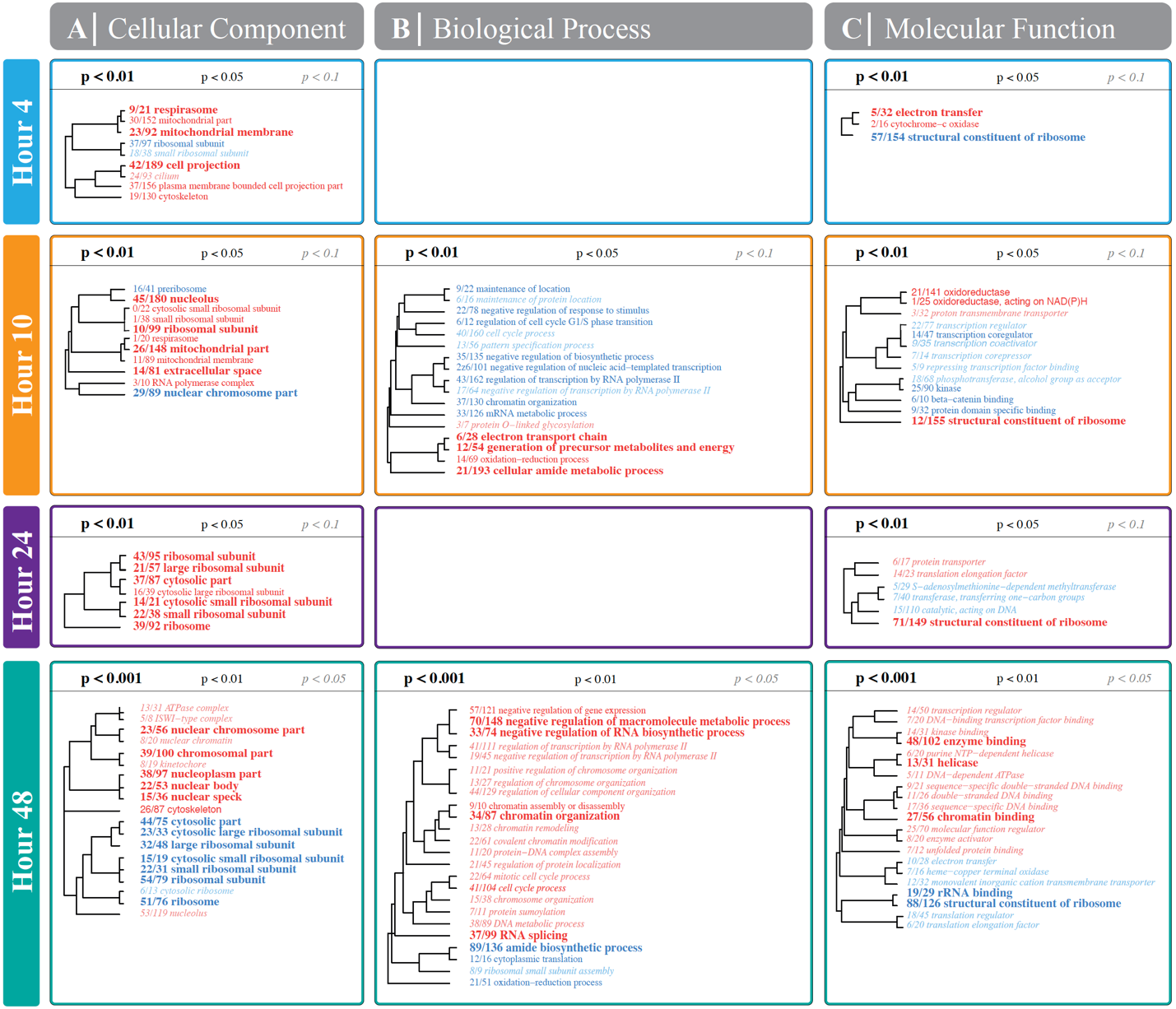
**

**Supplemental Tables**

Table S1 | Characteristics of seawater used for larval culturing (12 days) during the 16 d experiment (Experiment I). Values of pH are reported (from left) as the nominal treatment target values, the actual values for new seawater added to cultures, and the values recorded immediately before regular seawater changes. TA, total alkalinity; ΩAr, saturation state of aragonite; pCO2, partial pressure of carbon dioxide.


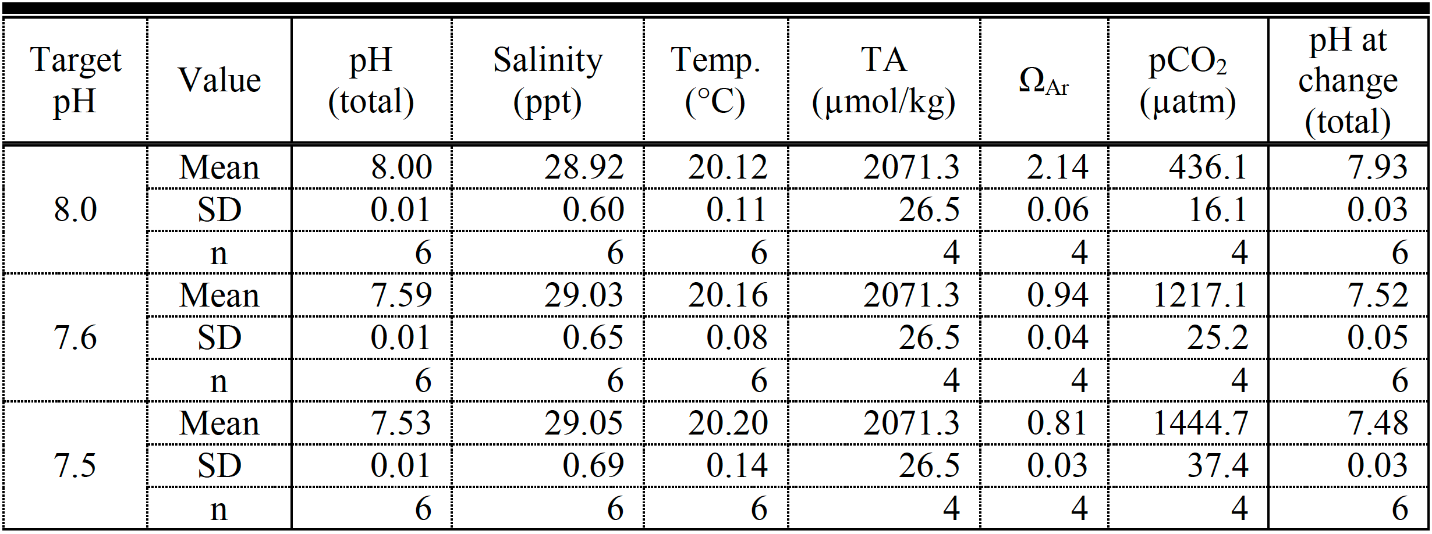


Table S2 | Characteristics of seawater used during the 48 h experiment (Experiment II). Values of pH are reported (from left) as the nominal treatment target values, and the actual values for new seawater added to cultures at the beginning of the experiment (0 h) or measured in 4 replicate cultures after 4, 10, 24, and 48h. TA, total alkalinity; Ω_Ar_, saturation state of aragonite; pCO_2_, partial pressure of carbon dioxide.


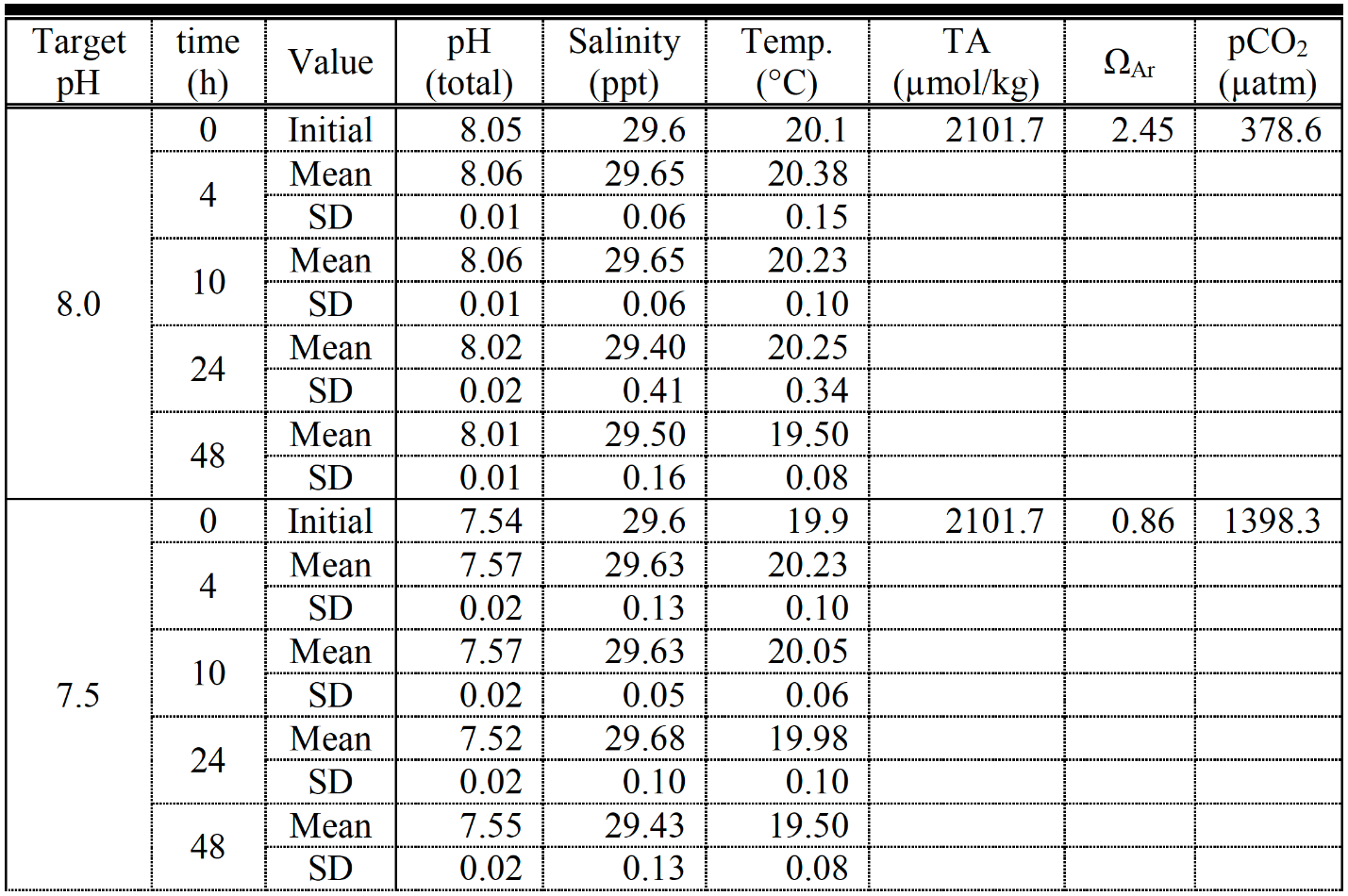


Table S3 | Summary of RNA libraries for the 16-d experiment (Experiment I), including raw single-end reads, trimmed reads, mapped counts, and mapping efficiencies (%). Samples found to be outliers during gene expression analyses and excluded from subsequent analyses are colored red.


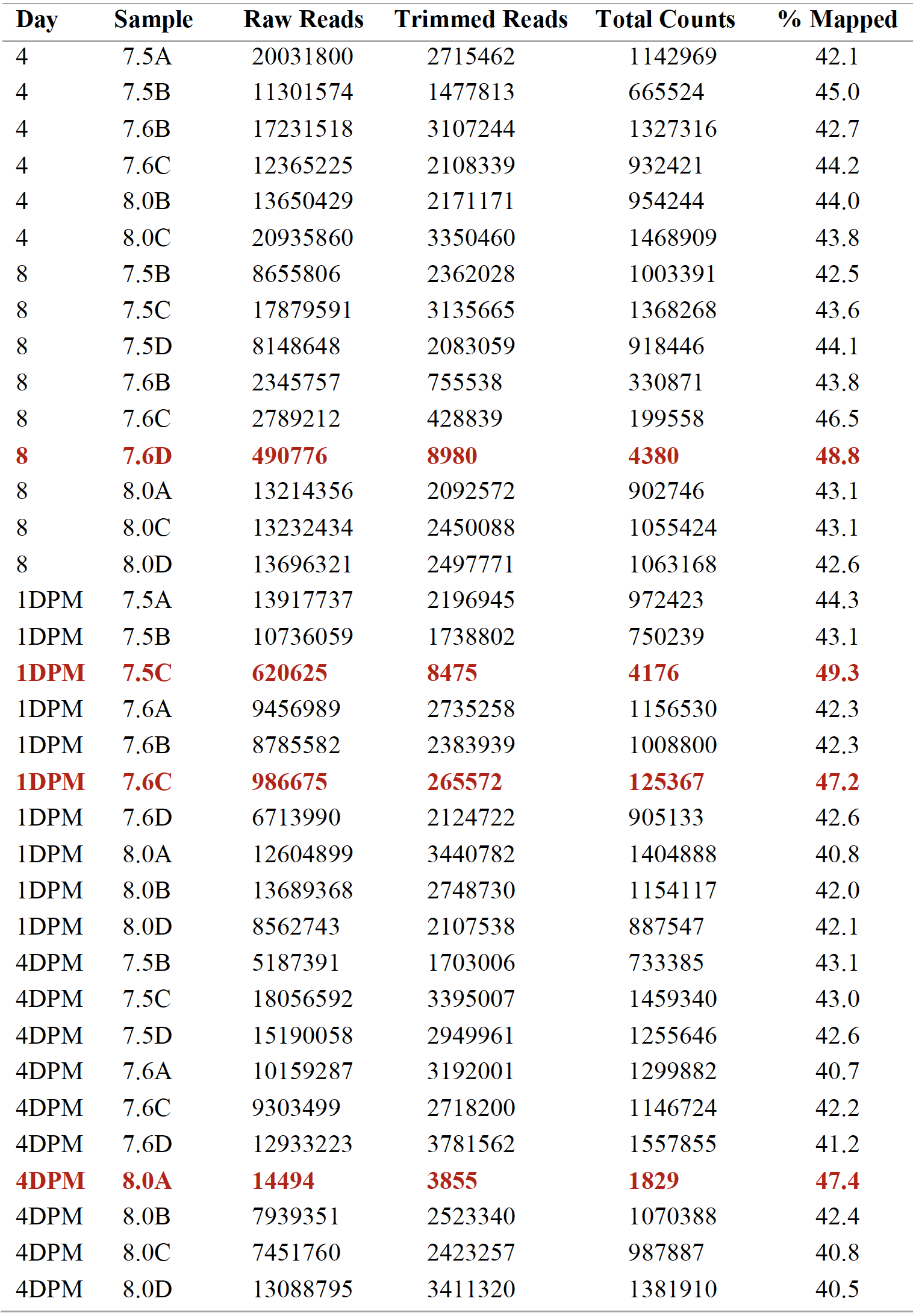


Table S4 | Summary of RNA libraries for the 48-h experiment (Experiment II), including raw single-end reads, trimmed reads, mapped counts, and mapping efficiencies (%). Samples found to be outliers during gene expression analyses and excluded from subsequent analyses are colored red.


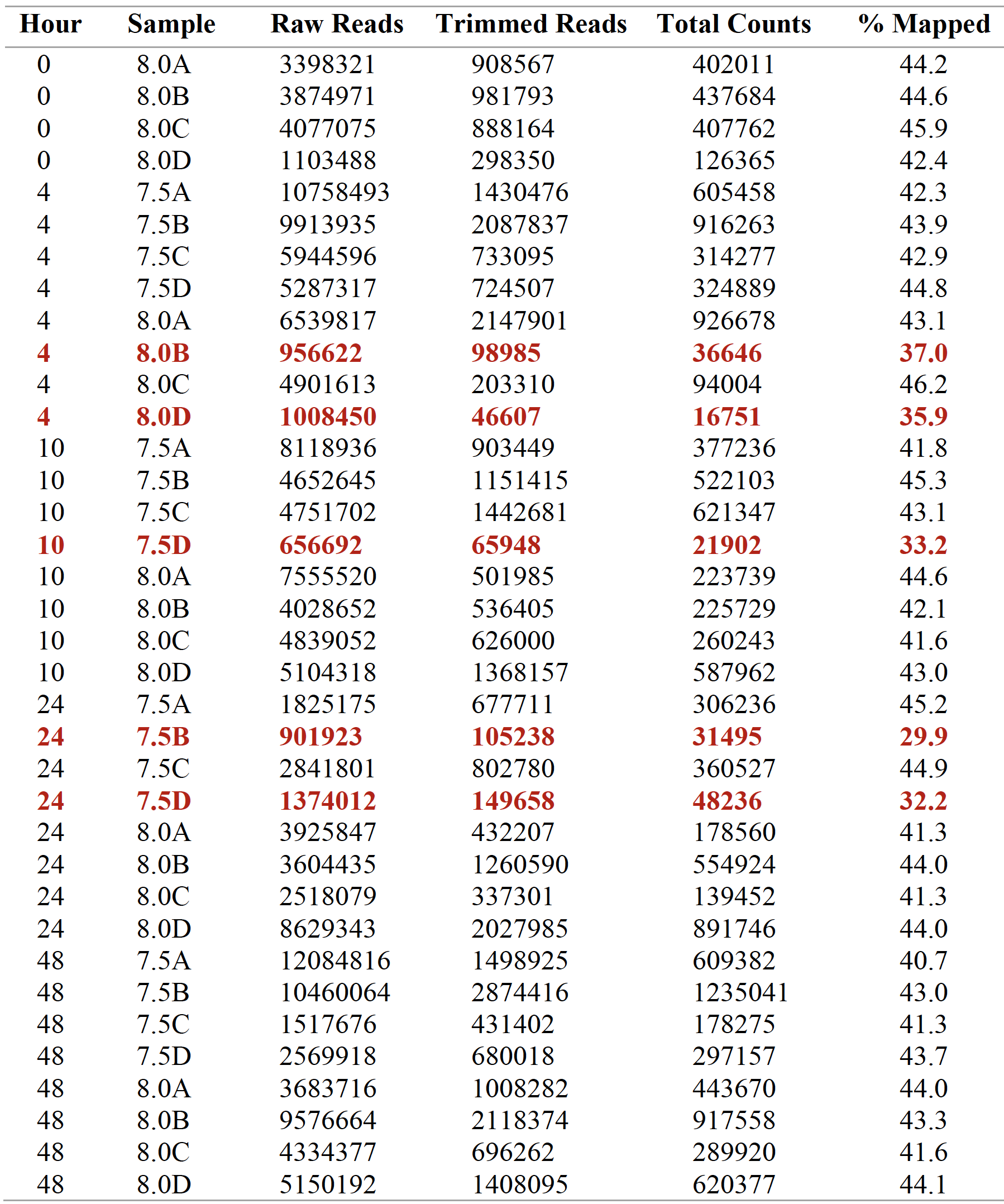

Supplement: Supplementary file 1 [file Data_Sheet_1.docx]
